# Two-phase numerical model for thermal conductivity and convective heat transfer in nanofluids

**DOI:** 10.1186/1556-276X-6-239

**Published:** 2011-03-21

**Authors:** Sasidhar Kondaraju, Joon Sang Lee

**Affiliations:** 1 Department of Mechanical Engineering, Yonsei University, Seoul, Korea

## Abstract

Due to the numerous applications of nanofluids, investigating and understanding of thermophysical properties of nanofluids has currently become one of the core issues. Although numerous theoretical and numerical models have been developed by previous researchers to understand the mechanism of enhanced heat transfer in nanofluids; to the best of our knowledge these models were limited to the study of either thermal conductivity or convective heat transfer of nanofluids. We have developed a numerical model which can estimate the enhancement in both the thermal conductivity and convective heat transfer in nanofluids. It also aids in understanding the mechanism of heat transfer enhancement. The study reveals that the nanoparticle dispersion in fluid medium and nanoparticle heat transport phenomenon are equally important in enhancement of thermal conductivity. However, the enhancement in convective heat transfer was caused mainly due to the nanoparticle heat transport mechanism. Ability of this model to be able to understand the mechanism of convective heat transfer enhancement distinguishes the model from rest of the available numerical models.

## Background

The thermal conductivity of thermofluid plays an important role in the development of energy-efficient heat transfer equipment. Passive enhancement methods are commonly utilized in the electronics and transportation devices, but the thermal conductivity of the working fluids such as ethylene glycol (EG), water and engine oil is relatively lower than those of solid particles. In that regard, the development of advanced heat transfer fluids with higher thermal conductivity is in a strong demand.

To obtain higher thermal conductivity, numerous theoretical and experimental studies of the effective thermal conductivity of solid-particle suspensions have been conducted dated back to the classic work of Maxwell [1]. The key idea was to exploit the very high thermal conductivity of solid particles, which can be hundreds and even thousands of times greater than that of the conventional heat transfer fluids such as ethylene glycol and water, but most of these studies were confined to suspensions of millimeter- and micrometer-sized particles [2,3]. Although such suspensions show higher thermal conductivity, they suffer from stability problems. In particular, particles tend to settle down very quickly and thereby causing severe clogging [4].

Unlike macro- and microparticles suspended in fluid, applications of nanoparticles provide an effective way of improving heat transfer characteristics of fluids. Particles, which are smaller than 100 nm in diameter exhibit properties different from those of microsized particles. It was demonstrated that nanofluids are extremely stable and exhibit no significant settling under static conditions [4,5]. From previous investigations [6-11], it was also observed that nanofluids exhibit substantially higher thermal conductivity even at very low volume concentrations (*Φ *< 0.05) of suspended nanoparticles.

Ever since it was observed that nanofluids showed an improved thermal conductivity, researchers have tried to develop numerical models to predict and understand the heat transfer mechanism in nanofluids accurately. Bhattacharya et al. [12] and Jain et al. [13] performed Brownian dynamic simulations to predict the thermal conductivity enhancement in nanofluids. Xuan and Yao [14] developed a lattice Boltzmann model to investigate the nanoparticle distribution in stationary fluid. Evans [15] and Sarkar and Selvam [16] have used molecular dynamics simulations to predict the thermal conductivity in nanofluids. Molecular dynamics simulations were performed at very small volume fractions or in highly idealized conditions and thus could not be validated with the experimental data. Simulation of naturalistic data would have necessitated a large computational power which is beyond the scope of current computers. To avoid this, the Brownian dynamics simulations omit fluid molecules and add the effect of hydrodynamic interactions by including position-dependent interparticle friction tensor. The above models can only be used to simulate the still fluid conditions and cannot be used to predict the convective heat transfer enhancement in nanofluids. To predict the convective heat transfer in nanofluids, Maiga et al. [17] performed numerical simulations using a single-phase Navier-Stokes model. The physical properties of nanofluids (density, thermal conductivity and viscosity) were predicted by assuming that the nanoparticles were well dispersed in the base fluid. The model cannot explain the mechanism of convective heat transfer enhancement in nanofluids because of the fact that the model is based on single-phase flow assumption. In the present study, a two-phase model is being considered. In this model, fluid properties are modified due to the dispersion of particles in the fluid medium and due to the interfacial interaction between particles and fluid. Thus, the need of correlation equations for predicting the change in fluid properties due to the presence of nanofluids can be evaded.

## Mathematical model

In the present study, an Eulerian-Lagrangian two-phase flow model is discussed, and the model is used to predict thermal conductivity and convective heat transfer enhancements in nanofluids. The model also gives an insight into the mechanism of heat transfer enhancements. The numerical model used in the present study solves for multiphase Navier-Stokes equations, where fluid phase is solved in Eulerian reference frame and particle phase is solved in Lagrangian reference frame. A brief overview of the model is presented in this paper. Readers are referred to S Kondaraju et al. [18] detailed information on the model.

In the Lagrangian frame of reference, the equation of motion of nanoparticle and time-dependent particle temperature equation are given by,(1)(2)(3)

Dispersion of nanoparticles was modeled by applying hydrodynamic drag force (*F*_Di_) [19], Brownian force (*F*_Bi_) [20], thermophoresis force (*F*_Ti_) [21] and van der Waals force (*F*_Vi_) [22] in the nanoparticle momentum equation. The coagulation of nanoparticles was also controlled by the van der Waals force acting on the adjacent nanoparticles. A cutoff distance of 0.2 nm was implemented in calculation of the van der Waals force. When the distance between the particles is less than the cutoff distance, particles were modeled to coagulate into one sphere with diameter equal to the summation of diameters of two coagulated particles. *x_i_^n ^*and *v_i_^n ^*are the instantaneous particle position and velocity of the *n*th particle, respectively. Subscript *i *represents the tensor notation. *τ*_T _is thermal response time of the particle and given as . *k*_f_, *d*_p_, *c*_p _and *ρ*_p _are the thermal conductivity of the base fluid, diameter, specific heat and density of the particle, respectively. **Nu **is the Nusselt number. *θ*_f _is the fluid fluctuation temperature in the neighborhood of the particle and *T*_p _is the temperature of the particle. It should be noted that in the present coagulation model the volume of coagulated particles is greater than the volume of particles when they coagulate in a real world situation (due to the assumption that two coagulated particles have a diameter equal to the summation of diameters of the two particles). However, the maximum increase in the volume concentration over time has been calculated and has been found to be of negligible amount to make any significant difference to the present results (see Appendix for the calculation).

Time-dependent, three-dimensional Navier-Stokes equations are solved in a cubical domain with the periodic boundary condition. The non-dimensional equations for fluid can be expressed as(4)(5)(6)

The cap '' is used in Equations 4-6, indicating that the values used here are non-dimensionalized. This model, which is often called as homogeneous thermal convection model assumes that the temperature field can be decomposed into the fluctuating part  subjected to periodic boundary conditions and the constant mean part *T***_o_**.  in Equation 6 denotes the mean temperature gradient in the *x*_2 _direction, which effectively acts as a source term for the fluid temperature field. The non-dimensional value of  is taken as 1.0 in the present simulations. Other parameters used in Equations 4, 5 and 6 are as follows: *u *is the velocity of the fluid, *p *is the pressure field, Re is the Reynolds number and Pr is the Prandtl number. Subscripts *i *and *j *represent tensor notations; and subscripts ',*i*' and ',*j*' represent differentiation with respect to *x_i _*and *x_j_*, respectively. *Q *is the linear forcing applied in the momentum equation to obtain a stationary isotropic turbulence. *F*_pi _[23] in Equation 4 is the net force exerted by the particles on fluid and *q*_2*w *_in Equation 6 is interfacial interaction between particles and liquid, which is modeled by addition of a temperature source term to the fluid temperature equation. It arises because of the convective heat transfer to and from the particle to fluid. In this model, *q*_2*w *_acts as a coupling term to couple particle temperature source to the fluid temperature equation. This coupling term is calculated by applying the action-reaction principle to a generic volume of fluid (here considered as a grid cell) containing a particle. In this paper, the term *q*_2*w *_is mentioned as a two-way temperature coupling term, and the effect of heat transport between particles and base fluid is called nanoparticle heat transfer. The equation for this coupling term is given as .

While performing the simulations of thermal conductivity, fluid is initially considered to be at still condition and constant temperature of 300 K. Motion of fluid and change in fluid temperatures occur due to simultaneous interactions of particle dispersion and particle heat transport with the fluid medium. The value of *Q *is considered to be 0 for the simulations carried out to study the thermal conductivity of nanofluids. For the simulations considering the study of convective heat transfer, a stationary isotropic fluid state is obtained at Taylor's Reynolds number of 33.01. Taylor's Reynolds number is calculated using Taylor's microscale length as the characteristic length. Taylor's microscale length (λ) is the largest length scale at which fluid viscosity significantly affects the dynamics of turbulent eddies. Taylor's microscale length (λ) is given as *λ *= (15*ν*/*ε*)^1/2^*u*', where *ν *is fluid viscosity, *ε *is fluid dissipation and *u*' is mean velocity fluctuations. Taylor's Reynolds number of 33.01 used in this simulation is equivalent to pipe flow Reynolds number of 5,500, and thus being turbulent, flow is chosen for this simulation. Simulating a higher Reynolds number at present is difficult due to an increase in thermal dissipation with an increase of Reynolds number, which will thus demand a very fine grid. The linear forcing coefficient used to maintain stationary turbulence is *Q *= 0.0667. The Prandtl number for all the simulations is taken as 5.1028, which is the Prandtl number of water at 300 K.

## Results

**Figure 1:**
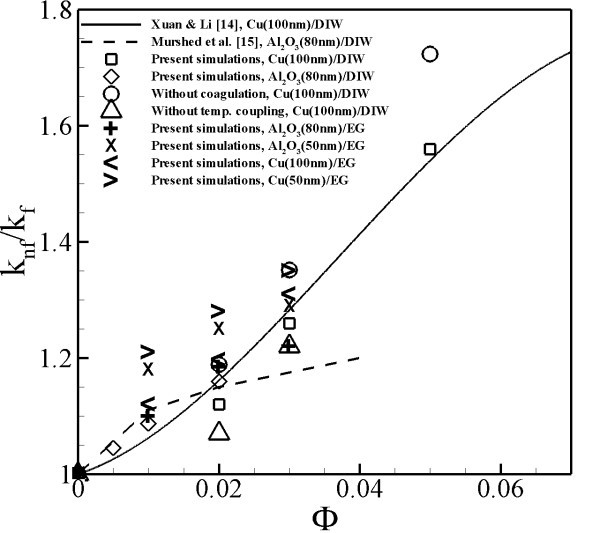
**Effective thermal conductivity of nanofluids**. Effective thermal conductivity of nanofluids at different volume fractions.

To validate the model, simulations were performed using the Cu(100 nm)/DIW **(distilled water) **and Al_2_O_3_(80 nm)/DIW nanofluids at different volume fractions. The turbulent thermal conductivity, which is the change in the conductivity of turbulent flow which is caused by the change of diffusivity of the flow, was determined by the equation [24], where *θ *is the fluctuation of temperature. The effective thermal conductivity of the nanofluid was then calculated as *k*_nf_/*k*_f _= (*k*_T _+ *k*_f_)/*k*_f_, where *k*_f _is the thermal conductivity of the fluid. The numerical data of present simulations is compared with the experimental data obtained by Xuan and Li [25] and Murshed et al. [26] (Figure 1). For the better understanding of the simulated results, values of the effective thermal conductivity of all the simulated nanofluids have been tabulated in Table 1. The calculated effective thermal conductivity values were observed to be in good agreement with the experimental data. The simulations underpredicted the effective thermal conductivity at 0.02 volume fraction for Cu(100 nm)/DIW nanofluid. A possible reason for this underprediction can be the discrepancy in prediction of the coagulation of particles in the present simulations, compared to the experiments. The values of effective thermal conductivity for the 0.03 and 0.05 volume fraction cases in the present simulations were closer to the experimental values. It can be observed that the values of Al_2_O_3_(80 nm)/DIW nanofluids show higher effective thermal conductivity at lower volume fractions in comparison with the effective thermal conductivity of Cu(100 nm)/DIW nanofluids. Cu(100 nm)/DIW nanofluids overtakes the effective thermal conductivity of Al_2_O_3_(80 nm)/DIW nanofluids at volume fraction above 0.02. Al_2_O_3 _being a non-metallic nanoparticle should have lower particle heat transport, which reduces the effectiveness of thermal conductivity enhancement at volume fraction greater than 0.02. However, at volume fractions lower than 0.02, higher effective thermal conductivity might be due to the smaller diameter of Al_2_O_3 _nanoparticles.

**Table 1:** Effective thermal conductivity of simulated nanofluids

Nanofluid Volume fraction	0.005	0.01	0.02	0.03	0.05
Cu(100 nm)/DIW			1.123	1.275	1.560
Cu(100 nm)/EG		1.135	1.191	1.313	
Cu(50 nm)/EG		1.220	1.273	1.362	
Al_2_O_3_(80 nm)/DIW	1.045	1.082	1.150		
Al_2_O_3_(80 nm)/EG		1.103	1.174	1.230	
Al_2_O_3_(50 nm)/EG		1.182	1.260	1.284	

Effective thermal conductivity of all simulated nanofluids is tabulated and shown here (computed values of effective thermal conductivity for simulations where the two-way temperature coupling and van der Waals force are neglected are not tabulated here).

In order to understand the effects of particle heat transport and coagulation of particles on thermal conductivity of nanofluids, simulations were performed for Cu(100 nm)/DIW nanofluids by neglecting two-way temperature coupling (*q*_2*w*_) and van der Waals interaction force (*F*_Vi_) one at a time. By neglecting two-way temperature coupling (*q*_2*w*_), we forbid the contribution of particles to the heat transfer enhancement in nanofluids and only calculate the contribution of enhancement due to the dispersion of particle in the fluid medium. Similarly, by neglecting the van der Waals interaction force (*F*_Vi_) we assume that the particles do not physically coagulate and observe the enhancement of heat transfer in nanofluids. Calculated effective thermal conductivity values are compared with the experimental data and simulation data where all the three parameters (i.e., particle dispersion, particle heat transport and coagulation of particles) are considered. When two-way temperature coupling is neglected, the results were found to be underpredicted by 4.45% for a 0.02-volume fraction of Cu(100 nm)/DIW nanofluid and by 3.62% for a 0.03-volume fraction of Cu(100 nm)/DIW nanofluid (Figure 1). The study suggests that both particle dispersions and particle heat transport have a contribution in the enhancement of effective thermal conductivity of nanofluids.

When the van der Waals force was neglected, the calculated thermal conductivity values are found to be overpredicted (Figure 1) as compared to experimental and simulation data where all the parameters are considered. Simulations, while neglecting the van der Waals force, were performed at 0.02, 0.03 and 0.05 volume fractions for Cu(100 nm)/DIW nanofluids. Overprediction of the calculated thermal conductivity is found to be increasing with an increase in the volume fraction. Difference between the calculated thermal conductivity values of with and without coagulation simulations is 6.13% for 0.02 volume fraction, 7.14% for 0.03 volume fraction and 10.47% for 0.05 volume fraction on Cu(100 nm)/DIW nanofluids. The study indicates that the coagulation of particles is one of the factors which are necessary to predict the thermal conductivity of nanofluids accurately.

Effect of different particle sizes and fluid medium on the effective thermal conductivity of nanofluids is also studied by performing simulations using Al_2_O_3 _nanoparticles of diameter 80 and 50 nm and Cu nanoparticles of diameter 100 and 50 nm by suspending them in the base fluid - EG. Simulations reveal that the size of nanoparticles has a great influence on the thermal conductivity of nanofluids. The smaller diameter of the particles will enhance the particle dispersion in the fluid medium which in turn can cause large disturbances in fluid and thus enhance the heat transfer rate of fluid. As can be seen from Figure 1 thermal conductivity of Al_2_O_3 _and Cu nanofluids increases dominantly when 50 nm particles are suspended in EG when in comparison with 80 or 100 nm particles. We have previously found that the decrease in size of nanoparticles leads to an increase in the particle dispersions and particle heat transport in the nanofluids which thus causes an increase in the effective thermal conductivity [18]. The figure also shows that with both DIW and EG base fluids, the thermal conductivity of nanofluids increases with increase in volume fraction. However, for a given volume fraction, it is observed that the thermal conductivity ratio enhancement is higher in EG. This behavior was consistently observed in both Cu and Al_2_O_3 _nanofluids. The reason for observed higher enhancement of thermal conductivity ratio in EG nanofluids could be due to the fact that the thermal conductivity of EG is low and thus the ratio of *k*_nf_/*k*_f _becomes larger.

The overall study of the thermal conductivity of nanofluids using the present model indicates a significant change in the effective thermal conductivity of nanofluids. Metallic nanoparticles were found to be more effective in enhancing the thermal conductivity of nanofluids. This could be due to stronger particle heat transport mechanism in metallic nanofluids. The study of different fluids indicates that nanoparticles, when suspended in EG, were more effective in enhancing the thermal conductivity of nanofluids. As the size of the nanoparticle decreases, the effective thermal conductivity of nanofluids was observed to be significantly enhanced. Simulations when performed by neglecting particle heat transport mechanism showed that the values of effective thermal conductivity are underpredicted, thus suggesting that both particle dispersion and particle heat transport have an effect on the enhancement of the effective thermal conductivity. Coagulation of particles is found to have a negative effect on the effective thermal conductivity enhancement. However, the simulations suggest that it is necessary to include van der Waals force in the numerical models to be able to accurately predict the thermal conductivity of nanofluids.

With the knowledge gained from the study of thermal conductivity of nanofluids, we included the terms particle dispersion, particle heat transport and coagulation of particles in our simulations of convective heat transfer in nanofluids. The study is more significant due to the fact that convective heat transfer of fluid has more practical applications. Also, though numerous simulations were performed to study the convective heat transfer enhancement in nanofluids, to our best knowledge the mechanism of heat transfer enhancement was not discussed by other researchers. We were interested in understanding the mechanism of heat transfer. An important question that lies ahead of us is if the particle dispersion of nanoparticles in fluid medium has a significant effect in the enhancement of the convective heat transfer in nanofluids.

**Figure 2:**
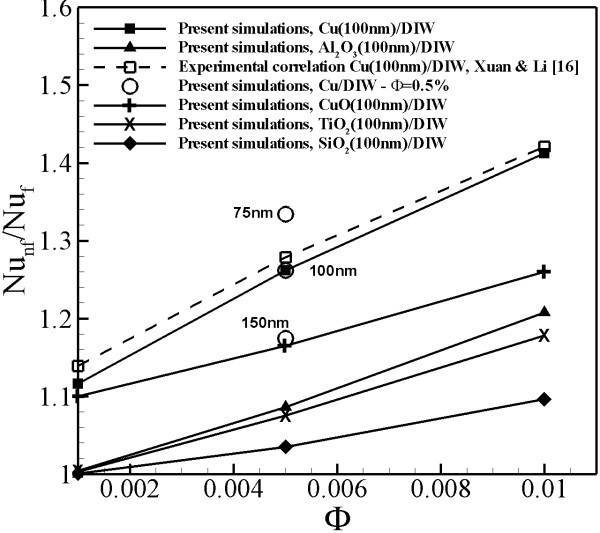
**Effective Nusselt number of nanofluids**. Effective Nusselt number for nanofluids at different volume fractions and particle diameters are shown.

In order to verify our model and also study the effect of different nanoparticle suspensions and size of nanoparticles on convective heat transfer of nanofluids, simulations were performed for Cu(100 nm)/DIW, Al_2_O_3_(100 nm)/DIW, CuO(100 nm)/DIW, TiO_2_(100 nm)/DIW and SiO_2_(100 nm)/DIW at 0.001, 0.005 and 0.01 volume fractions and for Cu(75 nm)/DIW, Cu(100 nm)/DIW and Cu(150 nm)/DIW at 0.005 volume fractions. The Nusselt number was calculated, using the formula , where *α *is the thermal diffusivity of fluid. The Nusselt number for Cu(100 nm)/DIW nanofluids at different volume fractions is compared with the experimental correlation (Figure 2) given in Xuan and Li [27] and is found to be in good agreement. The effect of volume fraction, particle material and particle size on the convective heat transfer can be observed in Figure 2. The Nusselt number increases with an increase in particle volume fraction and decreases with an increase in particle size. However, the enhancement of the Nusselt number is found to vary with the nanoparticle material suspended in the base fluid. For same volume fraction, it is found that the increase in Nusselt number is highest for Cu nanofluids and lowest for SiO_2 _nanofluids. The difference in the enhancement of the Nusselt number for different particle materials is due to the difference in their particle heat transport in nanofluids. As explained below, the particle heat transport plays the most important role in enhancement of convective heat transfer in nanofluids. Simulations of Cu/DIW nanofluids at 0.005 volume fraction for different particle sizes were performed to understand the effect of different particle sizes on the convective heat transfer enhancement. Nusselt number of Cu/DIW nanofluids at 0.005 volume fraction for different particle sizes is shown in Figure 2 with open circle 'O' symbols. The effective Nusselt number of different simulated cases is tabulated and shown in Table 2. It can be observed that with an increase of particle size, the Nusselt number of nanofluids decreases.

**Table 2:** Effective Nusselt number of simulated nanofluids

Nanofluid Volume fraction	0.001	0.005	0.01
Cu(100 nm)/DIW	1.120	1.271	1.425
Al_2_O_3_(100 nm)/DIW	1.005	1.072	1.207
CuO(100 nm)/EG	1.100	1.161	1.259
Ti0_2_(100 nm)/DIW	1.003	1.067	1.187
Si0_2_(100 nm)/EG	1.000	1.037	1.082
Cu(75 nm)/DIW		1.340	
Cu(150 nm)/DIW		1.164	

Effective Nusselt number of all simulated nanofluids is tabulated and shown here.

**Figure 3:**
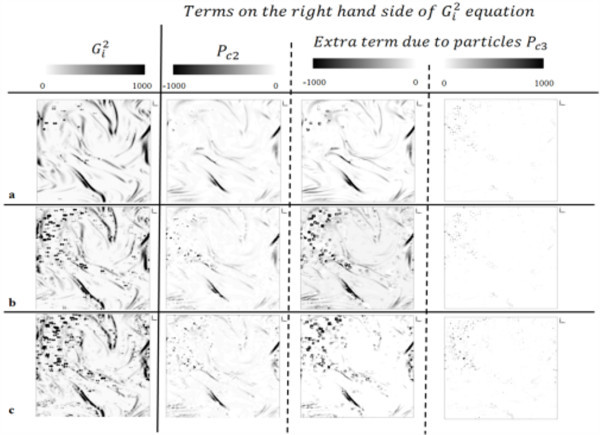
**Distribution of terms in square temperature gradient**. Distribution of , *P*_*c*2 _and negative and positive terms of *P*_*c*3 _are shown for Cu(100 nm)/DIW nanofluids at (a) *Φ *= 0.001, (b) *Φ *= 0.005 and (c) *Φ *= 0.01. Reprint from S. Kondaraju, E. K. Jin and J. S. Lee, Investigation of heat transfer in turbulent nanofluids using direct numerical simulations, 81, 016304, 2010. "Copyright 2010 by the American Physical Society."

To understand the mechanism of convective heat transfer in turbulent nanofluids, distribution of the production terms (*P*_*c*2 _and *P*_*c*3_) in transport equation of square temperature gradient () (Equation 7) and  are plotted for Cu(100 nm)/DIW nanofluids at 0.001, 0.005 and 0.01 volume fractions (Figure 3). *P*_*c*1 _, which is production caused by the mean temperature gradient in fluid temperature equation (Equation 6) was found to be 70 times smaller compared to *P*_*c*2_, which is production caused by the deformation of velocity field. Thus, it was assumed that the effect of *P*_*c*1 _on convective heat transfer is negligible and was not considered in further analysis. *P*_*c*3 _in Equation 7 is production caused by the particle heat transport effect on fluid medium, which is represented as *q*_2*w *_in Equation 6. Distribution of  shows an increase in the temperature gradients with an increase of particle volume fraction. However, the change in distribution of *P*_*c*2 _with change in particle volume fraction is found to be negligible. It suggests that the particle dispersions, which deform the fluid velocity, do not significantly affect the convective heat transfer rate in nanofluids. On the other hand, distribution of *P*_*c*3 _shows a significant difference at different particle volume fractions. Moreover, the high temperature gradients are found to be distributed in the regions of high magnitudes of *P*_*c*3_. It suggests a significant influence of particle heat transport on convective heat transfer of nanofluids.(7)

Simulations performed to study the convective heat transfer in nanofluids reveal that the convective heat transfer in nanofluids has significant influence from the kind of nanoparticles suspended in fluid medium. It was observed that the nanoparticles with higher heat transport rate show more enhancements in Nusselt number of nanofluids. The study of square temperature gradient and its production terms indicates that Equation 7, reveals that the particle dispersions in turbulent fluid, unlike in still fluid, do not significantly affect the heat transfer rate. It can be due to the presence of a large drag force on particles when the fluid is under turbulent conditions. The presence of a large drag force on particles in moving fluid nullifies the effect of other forces such as the Brownian force and thermophoresis force. However, all the simulations performed for the study of convective heat transport phenomenon in this paper, due to computational limitations, use nanoparticles with size 100 nm. We therefore have to study the effect of particle dispersions on convective heat transfer of nanofluids while using smaller sized particles, before a foregone conclusion can be made on the effect of particle dispersions.

## Conclusions

In this study, we have made an attempt to present a numerical model which can simulate and predict the thermal conductivity and also convective heat transfer in nanofluids. The model showed a good agreement with the experimental data. A wide range of particle sizes and nanoparticle materials used in the study also agree qualitatively with the results of previous researchers. A significant advantage of the present study is that it can help in understanding the mechanism of enhancement of thermal conductivity and Nusselt number in nanofluids.

## Competing interests

The authors declare that they have no competing interests.

## Authors' contributions

SK has carried out the simulations and participated in the analysis and interpretation of data. He also participated in drafting the manuscript. JSL conceived in the study and participated in data analysis and drafted the manuscript.

## Appendix

If the diameter of the two particles is considered as *d*_1 _and *d*_2_, an increase in the volume of particles (due to the method of coagulation in the present model) in the computational domain due to the agglomeration of two particles is given as follows.

The maximum increase in the volume of particles in the computational domain will be observed when all the particles coagulate into one single particle. The maximum number of particles (*n*) used in this study is 500,000 and the largest diameter of particles used is 100 nm. Thus, the maximum increase of volume of particles due to the present coagulation model is

When *n *= 500,000 and *d*_1 _= 100 nm,

The maximum increase in the volume of particles approximately equal to 15 × 10^-11^

Thus, it can be observed that the increase in the volume concentration of particles due to the present coagulation model will have a negligible effect on the simulated results.
